# The efficacy of behavioural activation treatment for co-occurring depression and substance use disorder (the activate study): a randomized controlled trial

**DOI:** 10.1186/s12888-016-0943-1

**Published:** 2016-07-08

**Authors:** Joanne Ross, Maree Teesson, Carl Lejuez, Katherine Mills, Sharlene Kaye, Kathleen Brady, Glenys Dore, Katrina Prior, Xanthe Larkin, Joanne Cassar, Philippa Ewer, Sonja Memedovic, Ivana Kihas, Sarah Louise Masters

**Affiliations:** National Drug and Alcohol Research Centre, University of New South Wales, Sydney, 2052 NSW Australia; NHMRC Centre for Research Excellence in Mental Health and Substance Use, University of New South Wales, Sydney, 2052 NSW Australia; Department of Psychology, College of Liberal Arts and Sciences, University of Kansas, Lawrence, 66045 KS USA; Department of Psychiatry, Medical University of South Carolina, 67 President St, Charleston, 29425 SC USA; Northern Sydney Drug and Alcohol Service, Herbert Street Clinic, Building 8, Royal North Shore Hospital, St Leonards, 2065 NSW Australia

**Keywords:** Activate, BATD-R, Behavioural activation, Depression, Substance use disorder, Randomised controlled trial

## Abstract

**Background:**

Epidemiological studies suggest that compared with the general population, mood disorders are up to 4.7 times more prevalent in substance dependent samples. Comorbid substance use disorder (SUD) and depression has been associated with a more severe and protracted illness course and poorer treatment outcomes. Despite this, the development and assessment of behavioural interventions for treating depression among individuals with SUDs have received little empirical attention. Behavioural Activation Treatment for Depression (BATD-R) is an empirically supported treatment for depression that has shown some efficacy among substance users. This paper describes the study protocol of a parallel, single blind, randomised controlled trial to determine the efficacy and feasibility of a modified version of the BATD-R (Activate) in reducing symptoms of depression and substance dependence among individuals in residential rehabilitation (RR) and opioid substitution therapy (OST).

**Methods/design:**

A sample of approximately 200 individuals with depressive symptomatology in treatment for SUD will be recruited from RR and OST services in New South Wales, Australia. Dynamic random allocation following minimisation methodology will be used to assign participants to one of two groups. The control group will receive treatment as usual (TAU), which will be the model of care provided in accordance with standard practice at participating RR and OST services. The intervention group will receive Activate, comprising 10 individual 60-min therapy sessions with a psychologist employed on the research team, in addition to TAU. Data collection will occur at baseline (pre-intervention), and 3-months and 12-months post baseline.

**Discussion:**

The association between depression and substance dependence has been well documented, yet practical and effective treatments are scarce. The findings of the present study will contribute significantly to understanding the types of programs that are effective in treating this comorbidity.

**Trial registration:**

This trial is registered with the Australian and New Zealand Clinical Trials registry, ACTRN12613000876796. Registered on 7 August, 2013.

**Electronic supplementary material:**

The online version of this article (doi:10.1186/s12888-016-0943-1) contains supplementary material, which is available to authorized users.

## Background

The frequent co-occurrence of depression and substance use disorders (SUDs) is well documented both in Australia and internationally [1–9], with epidemiological studies suggesting that affective disorders are up to 4.7 times more prevalent among substance dependent samples compared to the general population [10–12]. Particular cause for concern is the high prevalence of current major depression among treatment seeking substance users, with studies reporting rates as high as 20–55 % [2, 4, 13–15]. Despite evidence that depression is linked to poorer treatment outcomes [16–20], the development and assessment of behavioural interventions for depression among substance users has received little empirical attention.

### Impact of depression on treatment for drug dependence

Among substance users, depression has been associated with a more severe and protracted illness course, poorer social and occupational functioning, greater use of health services, an increased risk of post-traumatic stress disorder, higher risk of suicidal behaviours, and an increased risk of relapse in substance use problems [20–22]. The Australian Treatment Outcome Study (ATOS), a longitudinal study of treatment outcomes for heroin dependence, demonstrated that across a 3 year follow-up period clients who met DSM-IV criteria for major depression in the month preceding interview were more likely to be using heroin and other drugs, to be heroin-dependent, sharing needles, experiencing injection-related health problems, engaging in crime and were in poorer physical and mental health than non-depressed clients [20]. Depression was the only consistent predictor of poorer treatment outcome across the 3 year period [20]. Clearly, co-occurring depression and SUDs presents a complex clinical challenge for treatment providers that requires further empirical attention.

Depression and SUDs, when considered as separately occurring disorders, can be attributed to a number of environmental, genetic and neurobiological factors. In contrast, the mechanisms underlying these conditions as they co-occur are not well understood [23]. Four main hypotheses have been proposed to explain this comorbidity [24]. The self-medication hypothesis suggests that substance dependence occurs as a result of repeated substance use aimed at relieving the symptoms of depression. Secondly, depressive symptoms may develop as a direct result of taking substances or as part of withdrawal symptoms when substance use stops (e.g. substance-induced depression). Thirdly, depressive symptoms may develop in response to the lifestyle associated with using substances. Alternatively, both disorders may share common psychological or biological antecedents, thereby increasing the likelihood that they will co-occur. Regardless of which disorder came first, once co-occurring SUD and depression has been established, each may act to maintain or exacerbate the other [24].

### Current treatment protocols for co-occurring depression and SUDs

While treatment has traditionally been determined based on the temporal relationship between depression and SUDs (i.e., treating the disorder with the earlier onset first), there are practical difficulties in reliably diagnosing primary and secondary conditions [25]. It has been suggested that the primary/secondary distinction is immaterial once depression and SUDs have surfaced [26], and that the focus of treatment should be on the impairment and distress caused by symptoms, as opposed to diagnostic sub-type classification, or the model of aetiology [27].

There is some evidence, albeit limited, for the effectiveness of cognitive behavioural therapy (CBT) either alone or in combination with antidepressant use for the treatment of co-occurring depression and SUDs [28]. Unfortunately, however, only a handful of methodologically sound studies have been conducted to date, and these have focused on alcohol use outcomes, with limited reports on other substance use outcomes. While there is support for the use of CBT in the treatment of depression and co-occurring tobacco [29] and alcohol dependence [30], there are a number of difficulties when implementing depression focussed CBT treatment in a population dependent on a variety of licit and illicit substances, as outlined by Daughters and colleagues [31]. CBT focuses on complex cognitive techniques, which may be too cognitively challenging for chronic substance users with cognitive deficits and low education levels [32, 33]. Its time-consuming nature makes it difficult to incorporate into existing substance abuse treatments [34] and it requires a high level of training in complex therapy based treatments, which staff may not be able to deliver in traditional drug and alcohol treatment settings [35].

### Behavioural activation

A treatment that may be better suited for use among substance users is behavioural activation. According to this approach, symptoms and behaviours characteristic of depression arise when susceptible people experience problems that significantly reduce their ability to gain positive reward from their environment [36]. Behavioural activation is a structured treatment for depression designed to activate clients in ways that are tailored to increase rewarding experiences in their lives [36]. It is an action oriented approach that requires between session practice by clients, with therapists and clients working collaboratively to develop activation tasks for clients to complete outside the session and trouble-shoot obstacles that may arise. Empirical evidence suggests that behavioural activation therapy is just as effective as CBT [37, 38], and is more time efficient and less complex when compared to most other treatments for depression [39]. Behavioural activation therapy has been shown to be an efficacious, cost-effective alternative to cognitive therapy and antidepressant medication [40, 41].

Behavioural Activation Treatment for Depression (BATD) [39] is a brief manualised treatment which employs activity scheduling and other behavioural techniques within a framework applying matching law [42] to understand depression. This law suggests that depression persists when (a) reinforcement available for non-depressed (healthy) behaviour is low or non-existent, or (b) depressed (unhealthy) behaviour provides a relatively high degree of reinforcement [39]. Depression is therefore seen as the result of both decreased reinforcement for non-depressed, healthy behaviours, and increased reinforcement for depressed behaviours [43]. The structured BATD protocol seeks to increase non-depressed behaviour by providing exposure to the positive consequences of healthy behaviour, and has shown promise in treating depression among substance users [31, 44]. The manual was recently revised ([BATD-R; [45]), to improve delivery and patient acceptability.

### Evidence base for BATD

Lead developers of the manual, Lejeuz and Hopko, first pilot tested BATD among psychiatric inpatients suffering depression, and yielded a large pre-to-post treatment effect size (*d* = .73) for depression symptoms [46]. BATD gained additional support from three small randomised controlled pilot trials [31, 44, 47]. Daughters and colleagues (2008) added a behavioural intervention for depression (BATD) to inpatient treatment for illicit drug use (Lets Act!; *N* = 44) [31]. Those who received the Lets Act! intervention had significantly greater improvements in depression at post-treatment and 2-week follow-up compared with standard care alone. A subsequent trial compared Lets Act! with a time matched control treatment (supportive counselling), and found that while both groups showed a significant reduction in depressive symptoms on completion of treatment, retention in drug treatment was significantly greater in the behavioural activation group [44]. No follow-up data was collected beyond treatment completion. Another randomised controlled trial (*N* = 68) involving adult smokers with mildly elevated depressive symptoms and seeking smoking cessation treatment, found that participants allocated to ‘Behavioral Activation Treatment for Smoking (BATS)’, which was adapted from BATD, reported greater smoking abstinence, and a larger reduction in depressive symptoms at 16- and 24-week follow-up, than those who received standard smoking cessation treatment [47].

While the results of these trials are promising, and suggest preliminary support for the feasibility, acceptability and efficacy of brief behavioural activation treatment for improving mood and substance use outcomes, further research is needed. BATD-R was initially developed for use in the United States (US) and its efficacy has not been assessed in drug treatment services outside the US, among substance users in outpatient drug and alcohol treatment settings, or over long-term follow-up (i.e., 12-months) in either residential or outpatient drug treatment settings. The three clinical trials conducted to date have also all used a group rather than individual format.

## Methods/design

### Trial aims & hypotheses

The primary aim of the proposed trial is to determine the efficacy of a modified version of BATD-R (Activate) in reducing depressive symptoms among individuals with SUDs who are currently undergoing Residential Rehabilitation (RR) or Opioid Substitution Therapy (OST). It is hypothesised that:

1. Participants who receive Activate will demonstrate greater reductions in symptom severity for (i) depression and (ii) substance dependence, 3- and 12-months post baseline, compared to those who only receive treatment as usual.
2. Activate will be a feasible approach to treating comorbid depression and substance dependence, as measured by treatment retention and client satisfaction.

The secondary aim of the trial is to investigate the impact of other factors that may moderate or mediate the Activate treatment response. Secondary outcome measures will include those related directly to the therapy (environmental reward, behavioural activation), and those concerned with mental health (anxiety symptoms, social phobia, borderline personality disorder, traumatic events, rumination, distress tolerance, suicide history and sleep).

### Trial design

The Activate study is a parallel, single blind, superiority randomised controlled trial. Participants will be randomly assigned to receive either Activate (*n* = 100) or treatment as usual (TAU; *n* = 100), stratified by treatment modality (RR and OST). Treatment as usual is the comparator group, as it is clinically important to determine whether Activate adds any benefit in terms of improved depression symptomatology and reduced dependence symptoms, beyond that gained from usual care (i.e. RR or OST). Separate randomisation schedules will be used for the two treatment modalities. To reduce the risk of bias, minimisation, a dynamic random allocation technique, will be completed by someone independent of the research team using Minim-Py [48]. Gender and depression severity have been identified as significant prognostic factors and minimisation will ensure classification of participants with respect to these variables. Depression severity will be categorised according to BDI-II score, as mild (14–19), moderate (20–28) or severe (29–63) [49]. Minimisation has been shown to have both theoretical and practical validity, in particular it is considered an effective randomisation tool in clinical trials of small to moderate sample size (approximately, *N* = 200–400) with multiple prognostic factors to be considered [50–52].

The National Health and Medical Research Council of Australia (NHMRC) provided a project grant (#10444625) enabling the Activate study to be conducted. The funding period was initially February 2013 to December 2015, but was extended to December 2016 to accommodate extension of the baseline recruitment phase. Ethics approval was obtained from the University of New South Wales Human Research Ethics Committee (HREC) (Ref No: HC13155), and the Northern Sydney Local Health District HREC (Ref No: HREC/12/HAWKE /404). A model of the information and consent form used is available as supplementary material (Additional file 1). The study has been prospectively registered in the Australian and New Zealand Clinical Trials Registry (ACTRN12613000876796, 7/08/13). Any changes to the trial protocol which may impact the conduct of the study, the benefit to the patient or patient safety, will require a formal amendment to the protocol. Approval for such amendments will be sought from the HRECs and the trial registry will be updated. See Table 1 for the WHO Trial Registration Data Set. Additional information regarding groups overseeing the trial, insurance coverage, and authorship guidelines can be found in the supplementary material (Additional file 1).

**Table 1:** Trial registration data set as recommended by the World Health Organization (WHO)

Data category	Information
Primary registry and trial identifying number	Australian and New Zealand Clinical Trials registry, ACTRN12613000876796
Date of registration in primary registry	7 August, 2013
Secondary identifying numbers	Universal Trial Number U1111-1142-2213
Source(s) of monetary or material support	National Health and Medical Research Council
Primary sponsor	National Drug & Alcohol Research Centre, University of New South Wales, Sydney, NSW 2052
Contact for public queries	Joanne Ross (j.ross@unsw.edu.au)
Contact for scientific queries	Joanne Ross National Drug & Alcohol Research Centre, UNSW, Sydney, Australia
Public title	Treatment for depression among people with substance use disorder: The Activate Study
Scientific title	The efficacy of behavioural activation therapy for co-occurring depression and substance use disorder: The Activate Study
Countries of recruitment	Australia
Health condition(s) or problem(s) studied	Depression and Substance Use Disorder
Intervention(s)	The intervention is Behavioral Activation Treatment for Depression (BATD-R), modified for use in outpatient and in-patient drug and alcohol treatment settings (Activate). Comparator: Treatment as usual i.e. Residential Rehabilitation (RR) or Opioid Substitution Treatment (OST).
Key inclusion and exclusion criteria	Inclusion criteria: a) 18 years of age or older b) Literate in English c) Willing to give locator information d) Entered RR within the last month or in OST for at least 3 months e) Endorse CIDI 3.0 depression screening criteria f) Score at least in the mild range on PHQ-9 g) Substance use in the month prior to interview Exclusion criteria: a) Active suicidality b) Active psychosis c) Organic or traumatic brain injury d) Not living in the greater Sydney metropolitan area e) Not living in the community in the month prior to baseline
Study type	Interventional. Allocation: randomised; intervention model: parallel assignment; Masking: the research officers assessing outcomes are blind to the allocation Primary purpose: Treatment
Date of first enrolment	12/08/2013
Target sample size	200
Recruitment status	Recruitment completed. Analysis ongoing.
Primary outcome(s)	1) Depression Severity - Beck Depression Inventory (BDI-II) score - Composite International Diagnostic Interview 3.0 (CIDI) assessment of Major Depression. 2) Substance Use Disorder - CIDI assessment of substance use disorder - Severity of dependence scale (SDS)
Key secondary outcomes	1) Treatment feasibility as assessed by treatment retention and client satisfaction (assessed using the Client Satisfaction Questionnaire on completion of the Activate intervention) 2) Factors that influence the efficacy of the modified BATD-R (Activate) including client and treatment characteristics (assessed at baseline, 3 and 12 months).

Date and version identifier: Issue date: 17 Feb 2016

Protocol amendment number: 01 Authors: KP, JR

Revision chronology:

00, 7 August, 2013 Original

01, 17 February, 2016 Amendment 01.:

Two exclusion criteria, i.e. d) not living in the greater Sydney metropolitan area and e) not living in the community in the month prior to baseline, were added, as omitted from the original list

Actual date that the first participant entered the study was added (12.08.13)

Date of last participant enrolment was changed from 3.12.14 to 26.02.15. The baseline recruitment phase was extended due to delays with ethics and to help boost recruitment

List of recruitment sites was updated

File updated to indicate that Ethics Approval was obtained from Northern Sydney Local Health District HREC on 4 December 2013

#### Sample size calculation

Power analysis on the primary outcome variables (i.e., severity of dependence and depressive symptoms) was conducted using Power Analysis and Sample Size software [53]. Based on previous research, including the longitudinal study of treatment outcomes for heroin dependence (ATOS; [20]), an exchangeable working correlation matrix and a within-subject correlation of ρ^1^ = .60 for dependence and ρ^1^ = .023 for depression was assumed. The proposed final sample size of 160 will allow for the detection of clinically meaningful differences between the treatment and control groups. Specifically, it will allow for the detection of a time-averaged difference of 3 in severity of substance dependence with a standard deviation of 5 (β = 99 % power; *α* = 0.05); a 15 % difference in the prevalence of substance dependence (β = 96 % power; *α* = 0.05); a difference of 5 in BDI-II scores with a standard deviation of 15 (β = 81 % power; *α* = 0.05); and a 15 % difference in the prevalence of major depression (β = 93 % power; *α* = 0.05). To allow for an expected attrition rate of 20 %, the initial sample will be 200, with the aim of recruiting 50 % of participants from RR and 50 % from OST.

Participants will be 18 years of age or older, literate in English, willing to give locator information, and must have been in RR for at least 1 week or OST for at least 3 months. RR clients must have used a substance (i.e., alcohol, methamphetamines, cannabis, cocaine, hallucinogens, illicit benzodiazepines, heroin or other opiates/opioids) at least four times in the month prior to detoxing, and OST clients must have used a substance at least four times in the past month. If alcohol is the only substance used, individuals need to have consumed four or more standard drinks on at least 4 days in the month before detoxing/past month. The depression screening criteria from the Composite International Diagnostic Interview 3.0 (CIDI; [54, 55]) must be endorsed, indicating that the individual has experienced symptoms of depression that persisted for 2 weeks or longer in the month before detoxing/past month, and these symptoms must be ongoing. Depression symptom severity will be assessed using the Patient Health Questionnaire (PHQ-9; [56]), with a score in the mild to severe range (i.e., 5–27) required to be eligible for the study.

Active suicidality, psychosis and organic or traumatic brain injury will exclude an individual from entering the study. Suicidality is assessed by the PHQ-9 during the screening interview. Participants are asked “in the past 2 weeks/2-weeks before detox, have you been bothered by thoughts that you would be better off dead.” If participants indicate that they have been bothered by these thoughts, the research officer then probes further, using questions taken from the Suicide Assessment Kit screener [57], to determine whether these thoughts are ongoing, how long they have been having the thoughts, how intense they are, how likely it is that they will act on these thoughts in the near future, whether they have ever attempted suicide previously, and if so, how recently. Individuals who have attempted suicide in the past 6 months and for whom nothing has changed (i.e., they have received no intervention, or nothing has changed in their circumstances to reduce their risk), will be excluded from the study, and given a referral for appropriate support. If the thoughts are intense or the participant deems it likely that they will act on them in the near future, they are asked about any current plans and prior attempts, and are referred to an appropriate staff member at the treatment service for ongoing support and management.

Suicidality is assessed further as part of the baseline interview. The BDI-II and CIDI 3.0 both ask questions about suicidal thoughts, with the CIDI also asking about plans and attempts [49, 55]. Additional questions about the lifetime number of suicide attempts and recency of the last attempt are also asked. In situations where suicidality is indicated, the research officer uses the additional probing questions described above to assess their current risk, and to determine whether there is a need for exclusion from the study and referral for additional support. It is possible for a person with a current plan and thoughts of suicide to be recruited to the study provided the risk is not deemed imminent, and the treatment service is made aware of the suicidal thoughts and plan.

As part of the screening questionnaire, potential participants are asked whether they have ever been diagnosed with bipolar, mania, or schizophrenia. If the response is yes, (or if the individual reports being prescribed antipsychotics during the baseline interview), the project psychologist will meet briefly with them and ask them whether they ever see or hear things that other people cannot see or hear, whether the hallucinations are believed to be drug induced, whether the hallucinations are current, and how these are managed. These individuals are also asked about having unusual thoughts or beliefs. The psychologist then makes a clinical decision about their eligibility for the study. Self-report of having received a diagnosis of traumatic brain injury, as ascertained during the screening interview, is also a reason for exclusion.

Exclusion from the study in all of the above cases is based on the individual’s inability to effectively engage with, and thus benefit from, the type of intervention offered. Decisions to exclude on the basis of suicidality, psychosis or traumatic brain injury will all be made in consultation with the project coordinator and/or a project psychologist prior to randomisation. Alternate referral for treatment will be provided where necessary. Additionally, for reasons of feasibility in providing the Activate intervention and contacting participants for follow-up, clients will be excluded if they do not live in the Greater Sydney Metropolitan, Central Coast or Hunter New England regions. To ensure that participants have been at liberty to use substances in the month prior to detox or past month, individuals not living in the community (e.g., incarcerated) during that period will be excluded.

### Study procedure

#### Sample site recruitment

Participants will be recruited from RR services and OST clinics in greater Sydney metropolitan area, the Central Coast, and Hunter New England Health regions. Management staff will be contacted by the project coordinator to determine their initial interest in being involved. A letter outlining the purpose of the trial, and what will be required of the agency should they choose to participate, will be sent to the services. On confirmation of intent to participate, the research team will meet with the staff to discuss logistics, and recruitment of study participants will commence.

#### Screening

Staff in the RR and OST services will inform clients that the University of New South Wales is conducting a study examining mood and substance use. They will direct those interested in hearing about the study and being screened for eligibility, to one of the project’s research officers who will be on-site to conduct the screening to help maximise recruitment. Informed written consent will then be sought by the research officers from eligible clients interested in participating, locator information to facilitate follow-up will be collected, and the baseline interview conducted. The research officers (honours graduates in psychology) have been trained in the assessment interview by the investigators (MT, JR and KM). Participants will be informed that they are free to withdraw from the study at any stage. Recruitment will occur in ten treatment services over a period of 18 months. The locations of the treatment services are listed in the Australian and New Zealand Clinical Trials registry.

#### Assessment occasions

**Fig. 1:**
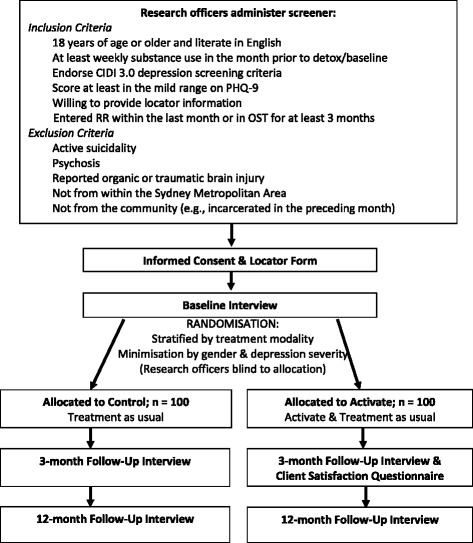
Flow Diagram of Trial Progression

The structured baseline interview will be administered by the research officers, followed by randomisation of participants to one of two groups; (1) TAU, or (2) Activate plus TAU (Fig. 1). The allocation sequence will be computer-generated by someone independent of the Activate team. The project coordinator will then inform the psychologist about the allocation, and they will notify the study participant. Follow-up interviews for the Activate and TAU groups will parallel each other, conducted at 3-months and 12-months post-baseline. Primary and secondary outcome measures will be assessed at all three assessment occasions, with the exception of client satisfaction, which will be assessed on completion or cessation of the Activate intervention. Follow-up interviews will be administered by the project’s research officers who will be blind to group allocation. The baseline and follow-up interviews are estimated to take 60–90 min to complete. Participants will be reimbursed AU$30 per interview for out of pocket expenses.

#### Locator information

A locator form will be used to record participant information that may be used to facilitate follow-up. This information will be collected at baseline and updated on both follow-up occasions. Consent to the study also provides permission for researchers to locate the participant by various means, such as social media, telephone directories, court lists, electoral rolls, and treatment services. These methods are based on those used in ATOS which achieved follow-up rates of 89, 80, 76, and 70 % at 3-, 12-, 24-, and 36-month follow-ups respectively [58].

#### Data management

To maintain confidentiality of participant data, all participant information will be stored in locked filling cabinets at the National Drug and Alcohol Research Centre, with access restricted to the research team. All records that contain names or other personal identifiers (such as locator forms and consent forms), will be stored separately from questionnaires, which will be identified by a participant identification number. All local databases will be secured with password protected access systems. Data entry will be conducted by the research officers. To maximise accuracy and consistency of data entry, a data dictionary will be used to define the variables and specify how they are to be coded. To promote data quality value range checks and consistency checks will be performed.

### Measures

#### Baseline and follow-up interviews

The baseline and follow-up interviews will examine demographics, drug use history, treatment history (for substance use and depression), and will use validated instruments to assess a) the primary treatment outcomes of depression and substance dependence, and b) secondary treatment outcomes related to the therapy and mental health. Demographic measures will include age, gender, level of education, employment status, main source of income, marital status, living arrangements, and prison history. A drug use history (including age of first use, and the number of days used in the last 30 and 90 days), will be obtained for heroin, other opiates, methamphetamine, cocaine, hallucinogens, antidepressants, benzodiazepines, alcohol, cannabis, inhalants and tobacco. Prescription drug use will also be recorded. Drug treatment and depression treatment history will assess current treatment(s), the age at which they first sought treatment, number of times they have commenced treatment, and the recency of these treatments. Information will also be obtained on the participants’ history of suicide, self-harm, and occasions where they lost consciousness as the result of a head injury.

#### Primary outcome measures

Depression: The severity of depression symptoms in the past 2 weeks will be assessed using the 21-item Beck Depression Inventory (BDI-II; [49]). Each item comprises a list of four statements arranged in increasing severity about a particular symptom of depression. Scores range from 0 to 63, with higher total scores indicating more severe depressive symptoms. Depression can then be interpreted as minimal (0–13), mild (14–19), moderate (20–28), or severe (≥29). The BDI-II has been widely used in both clinical and non-clinical studies, has shown good test-retest reliability (Pearson’s *r* = .73-.96), and high internal consistency (Chronbach’s α = .83-.96) [59]. A past month diagnosis of major depression will be ascertained by completing the CIDI 3.0 depression section, modified to assess the presence of symptoms in the past month [54, 55]. The CIDI 3.0 major depression module has been found to have moderate concordance with the Structured Clinical Interview for DSM (SCID) (area under the receiver operator curve; AUC = 0.75) [60].

Substance Dependence: Psychological components of dependence will be measured using the Severity of Dependence Scale (SDS; [61]). Each of the 5 items is scored on a 4-point scale, ranging from 0 to 3. A total score is obtained, with higher scores indicating a higher level of dependence. The inter-item reliability has been demonstrated across five drug using samples, with Cronbach’s alpha values ranging between 0.8 and 0.9 [61]. A diagnosis of substance dependence will be established using the CIDI 3.0, with the number of dependence criteria endorsed (0–7) being used as an indicator of severity [54]. The CIDI 3.0 substance use disorder module has been found to have moderate concordance with the SCID (Alcohol dependence, AUC = 0.72; Drug dependence, AUC = 0.62) [60].

Problematic alcohol use will be screened for using the 4-item CAGE questionnaire [62]. Scores range from 0 to 4, with scores greater than or equal to 2 considered to be clinically significant. The CAGE has demonstrated high test-retest reliability (*r* = 0.80–0.95), and adequate correlations (*r* = 0.48–0.70) with other screening instruments such as the Alcohol Use Disorders Identification Test (AUDIT) and Short Michigan Alcohol Screening Test (SMAST) [63].

#### Symptom monitoring and secondary outcome measures

The Patient Health Questionnaire-9 (PHQ-9) will be used in the initial screening interview, and then by therapists to monitor depressive symptoms among the intervention group [56]. The PHQ-9 is a nine-item screener for major depression and a measure of depression severity, which assesses the presence of each of the nine DSM-IV criteria for major depression in the preceding 2 weeks. Scores range from 0 to 27, as each of the nine items can be scored from ‘0’ (not at all) to ‘3’ (nearly every day) [56]. Major depression is indicated if 5 or more of the 9 depressive symptom criteria have been present at least “more than half the days” in the past 2 weeks, and 1 of the symptoms is depressed mood or anhedonia. The PHQ-9 has demonstrated excellent internal reliability (Chronbach’s α = 0.86–0.89), and test-retest reliability (*r* = 0.84) [56]. Criterion validity has been demonstrated in a sample of 580 primary care patients who were independently re-interviewed by a mental health professional. Receiver Operator Curve (ROC) analysis showed that the area under the curve for the PHQ-9 in diagnosing major depression was 0.95, suggesting that it discriminates well between persons with and without major depression [56].

Client satisfaction will be assessed after completion of Activate therapy using the Client Satisfaction Questionnaire, an 8-item scale designed to measure and assess client satisfaction with health and human services (CSQ-8; [64]). The CSQ-8 elicits the client’s perspective on the value of services received. Responses are given on a 4-point Likert scale. Scores range from 8 to 32, with higher scores indicating greater satisfaction. The CSQ-8 has demonstrated excellent reliability and internal consistency (Cronbach’s alpha 0.92 to 0.93) [64]. Secondary outcome measures, including those associated with behavioural activation therapy and mental health will be assessed at baseline, 3- and 12-month follow-up (Table 2).

**Table 2:** Schedule of assessments

Domain	Measure	Screening	Baseline Pre-allocation	3 month follow-up	12 month Follow-up
*Eligibility*					
Depression symptoms	Patient Health Questionnaire (PHQ-9) [56]	+	−	−	−
Depression screener	CIDI 3.0 Depression screener questions [54, 55]	+	−	−	−
*Primary outcome measures*					
Substance dependence	CIDI 3.0 Substance dependence (past month DSM-IV TR) [54] Severity of Dependence Scale (SDS) [61]	− −	+ +	+ +	+ +
Depression	CIDI 3.0 Major depression (past month DSM-IV TR) [54, 55] Beck Depression Inventory-II (BDI-II) [49]	− −	+ +	+ +	+ +
*Secondary outcome measures*					
Behavioural activation	Behavioural Activation for Depression Scale Short Form (BADS-SF; [70])	−	+	+	+
Anxiety	Beck Anxiety Inventory (BAI; [71])	−	+	+	+
Social phobia	CIDI 3.0 Social Phobia (past month DSM-IV-TR) [54] Liebowitz Social Anxiety Scale [72, 73]	− −	+ +	+ +	+ +
Traumatic events & trauma symptoms	PTSD trauma checklist from the CIDI 3.0 [54] and the Post-Traumatic Stress Disorder Checklist – Civilian Version (PCL-C; [74])	-	+	+	+
Borderline Personality Disorder	International Personality Disorders Examination (IPDE; [75])	−	+	+	+
Environmental Reward	Environmental Rewards Observation Scale (EROS; [76])	−	+	+	+
Rumination	Perseverative Thinking Questionnaire (PTQ; [77, 78]) and Ruminative Response Scale (RRS; [79])	−	+	+	+
Distress Tolerance	Distress Tolerance Scale (DTS; [80])	−	+	+	+
Sleep disturbance	Pittsburgh Sleep Quality Index (PSQI; [81])	−	+	+	+
Chronic physical conditions	As outlined by the Australian National Health Priority Area [82, 83]	−	+	+	+

#### Intervention

The trial will use a modified version of the BATD-R manual (Activate), which involves 10 individual, weekly 60-min sessions, with a psychologist from the research team. The 10-session format allows for the inclusion of topics deemed important for the target population, and there is evidence that clinically significant improvements in depression symptoms can be achieved within this period [45]. The BATD-R manual was modified to include psychoeducation about the relationship between substance use and depression. To assist in establishing treatment goals in relation to their substance use, some motivational interviewing techniques were incorporated. Namely, this involved asking questions to elicit change talk, such as, ‘*What would you like to be different about your current situation?’* Other additions include the provision of a list of behavioural techniques for managing craving [65], and a simple breathing exercise. Given the high levels of psychiatric comorbidity among substance users in drug treatment [66, 67], self-soothing and grounding techniques have been included for managing emotional distress, and defusion exercises for the management of unhelpful thoughts. Participants may choose to withdraw from the intervention at any time, but will still be contacted for follow-up assessment, unless they have withdrawn from the study.

To encourage treatment attendance participants in Activate will receive a reminder call the day prior to each session to confirm their appointment for the following day and appointment times will be highly flexible throughout the treatment program, with the option to reschedule appointments if required. Therapists will keep logs of clients’ attendance at sessions.

The use of project therapists, as opposed to existing clinicians within treatment agencies, has been chosen to minimise the risk of contamination of the control group. In order to avoid the outcome of Activate being attributed to a single therapist bias, two clinical psychologists will be employed. Both psychologists will receive extensive training in treatment delivery, provided by the lead developer of BATD-R, Carl Lejuez, and will be supervised by a senior clinical psychologist with experience in the treatment of depression and substance use. The Activate treatment manual will be taken into each session by the therapist to ensure all proposed areas are covered. The therapist will also complete a session checklist, with any deviations from the therapy manual recorded in the clinical notes kept for each session. Weekly clinical supervision will be held with the chief investigators, where session checklists will be monitored. In addition, all treatment sessions will be audio-recorded to allow for assessment of treatment fidelity.

### Overview of treatment

Session One covers the procedural aspects of treatment such as conditions of confidentiality, the recording of sessions, suicide risk assessment as well as guidelines for treatment and emphasising the importance of attending each weekly session. Session One also provides psychoeducation about the role of substance use in depression, explores the participant’s current situation and their ideal life, reviews the treatment rationale as well as introducing behaviour monitoring. The first session introduces the weekly homework assignment which is repeated each subsequent session. This involves the monitoring of activities on an hourly basis with the participant also providing ratings on the enjoyment and importance of each activity on a numerical scale from 1 to 10, as well as a rating of their overall daily mood from 1 to 10 (1 being low and 10 being high).

Session Two begins with the revision of substance use, depression symptoms and suicide risk, followed by a detailed review of the daily monitoring forms completed that week. This review occurs in all subsequent sessions. Enjoyment and importance ratings are considered for each activity and participants explore any difficulties that they had completing monitoring forms. The treatment rationale is reviewed prior to introducing the concept of life areas (relationships; education/career; recreation/interests; mind/body/spirituality and daily responsibilities). The majority of the session focuses on exploring the participant’s values within each of the life areas and generating activities that are associated with each life area. Finally, participants are introduced to a list of behavioural activities to assist in coping with cravings for substances. These include using delay and distract techniques, managing hunger and thirst, using controlled breathing techniques, imagery exercises such as “urge surfing”, seeking social support, and using positive self-talk.

During the third session, participants continue to generate activities that are value based for each life area. They then choose fifteen of these activities and rank them in order from easiest to hardest taking into account the level of expected enjoyment and importance of each activity. The participant is encouraged to include some activities that they are already engaged in. Session Three concludes with the introduction of the two brief behavioural techniques for managing emotional distress, these include a self-soothing exercise (where the client is asked to use their five senses to create more soothing moments in their day), and a breathing exercise where participants slow their breathing and think of the word “relax” when exhaling.

In Session Four participants revisit the activity list from the previous session and choose activities to schedule into their monitoring forms for the upcoming week. They progress from easier to harder activities and have the opportunity to reassess the order of activities throughout sessions. The process of selecting activities from their list occurs each session until the end of treatment. Participants are encouraged to choose between one and three new activities each week from a variety of different life areas. Session four ends with the introduction of a defusion exercise (e.g. using repetition or singing the thought).

Session Five introduces the concept of contracts with significant others. Participants are encouraged to select an activity that they have found difficult to complete and then select three friends or family members who may be able to assist them to complete this activity. The aim is to increase social support and increase enjoyment in completing activities. Sessions Six to Ten reinforce material previously introduced with a focus on activity selection and monitoring. In Session Six a simple grounding exercise is introduced at the end of the session (notice five things you can see, hear and feel). Similarly, at the end of Session Eight a basic mindfulness exercise is introduced for participants to practice.

Session Ten concludes treatment with a list of skills achieved and an opportunity to explore the management of potential setbacks. Throughout treatment, participants are encouraged to recognise patterns of depressed behaviour and the way in which engaging in enjoyable and important activities may impact their overall mood.

Prior to each session participants will complete the PHQ-9 [56] and be asked how many days they have used substances (alcohol, cannabis, benzodiazepines, heroin, other opiates, methamphetamine, and ‘other’) in the past week. This will enable therapists to monitor depressive symptoms and substance use throughout treatment, and help to identify unfavourable changes in symptoms. If concerned for the safety and wellbeing of the participant, the therapist will consult with the project coordinator, additional support services will be offered to the participant, and it will be determined whether or not ongoing therapy is appropriate for them. Although unlikely, any adverse events attributed to the intervention will be reported the HRECs. The participant information sheet also instructs participants to contact the project coordinator if they experience “significant distress as part of this study” and provides contact details for the ethics secretariat should they which to make a complaint about the conduct of the study.

#### Treatment as usual

The control group will receive the model of care provided in accordance with standard practice at participating RR and OST services, and will be delivered by agency staff. In addition to standard drug and alcohol treatment, clients in RR may receive additional treatment from case workers or in-house psychologists. Similarly, in addition to methadone or buprenorphine maintenance for managing heroin dependence, OST clients may also receive counselling for depressive symptoms, antidepressant medication where appropriate, and referral to mental health services when required. All treatment for depression received as a part of TAU will be recorded in participant interviews. These will be controlled for in comparative analysis of treatment outcomes.

### Statistical analysis

The proportion of screened clients entering the study will be determined. T-tests and chi-square analyses will be conducted to determine if those who did not enrol in the study were systematically different from those who did. T-tests and chi-square analyses will also be conducted to determine if there were any significant differences between the treatment and control groups at baseline. For outcome analyses, both intention-to-treat (i.e. analyses include all randomised participants, regardless of protocol adherence) and per-protocol analyses (i.e. where the intervention group only includes participants who received at least one session) will be conducted. Categorical and continuous measures of outcome will be examined using mixed or marginal longitudinal models.

To evaluate treatment fidelity, 10 % of each therapist’s sessions will be rated for compliance with the treatment manual. Interim descriptive analyses will take place at each assessment stage (including the baseline assessment), to assist with the monitoring of data collection and data entry. These analyses will be conducted by the research officers, who will not have access to the treatment allocation variable. Final stage analyses will be carried out after completion of the 12 month follow-up.

#### Dissemination of results

The research team intend presenting the findings of this trial at professional seminars, national and international conferences, and in manuscripts submitted for publication in peer-reviewed journals. Results will also be reported to the UNSW and Northern Sydney Local Health District HRECs. Only aggregated group data will be reported and no individuals will be identified.

## Discussion

This protocol presents the design of the Activate study, a randomised controlled trial, which seeks to evaluate the efficacy of a modified version of BATD-R (Activate) among individuals in treatment for SUDs. The primary aim of the study is to investigate the efficacy of the Activate intervention in comparison to treatment as usual, in reducing symptoms of depression and substance dependence. For the Activate intervention group, treatment retention, client compliance, and client satisfaction will be assessed. The secondary aim of the study is to examine whether other factors, including those associated with therapy (environmental reward, behavioural activation), and those concerned with mental health (anxiety and social phobia, borderline personality disorder, traumatic events, rumination, distress tolerance, and sleep), impact upon the Activate treatment response.

### Strengths and limitations

A strength of the Activate study is that it is the first randomised controlled trial to evaluate the efficacy of behavioural activation therapy for depression among individuals in both inpatient and outpatient substance abuse treatment settings, and in an individual format. Follow-up assessments will occur at 3- and 12-months post-baseline in both the Activate intervention group and the control (TAU) group, which will provide an opportunity to evaluate the long-term effects of Activate among this population for the first time. Primary outcomes of severity of depression and dependence will be assessed by research officers who will be blind to treatment allocation, using validated measures. The incorporation of some motivational interviewing techniques, a brief relaxation strategy, and psychoeducation about the role of substance use in depression targets the intervention more specifically for substance users. Defusion exercises, self-soothing and grounding techniques have been included to equip participants with skills for managing rumination and emotional distress, which are prevalent among this group [68], so they can attend to the BATD-R protocol more fully.

One limitation of the Activate study is excluding individuals who have active suicidality, self-harm or psychosis, and those who report organic or traumatic brain injury. This may be considered to reduce the generalisability of results to a real world sample. However, these exclusion criteria were based on the individual’s likely inability to effectively engage with, and thus benefit from the type of intervention offered. A potential challenge for the study will be retaining participants, as individuals with SUDs are traditionally very difficult to follow-up. To maximise follow-up rates, research officers will build good rapport with participants, obtain detailed contact information to help with locating participants, reinforce to participants the importance of conducting follow-ups, and financially compensate participants for the time required to complete the interviews (AU$30). These methods have been utilised in previous studies among substance users, which achieved commendable 12-month follow-up rates of around 80 % [20, 69].

## Conclusion

Integrated treatment is recommended in people with psychiatric illness comorbid with substance use problems. Yet, there are currently few evidence based treatment options for co-occurring depression and SUD. BATD-R is a manualised treatment that has shown promise among drug and alcohol residential rehabilitation clients [31, 44] and among psychiatric inpatients [46], but larger, longer term trials are needed to broaden the BATD-R evidence base. The Activate study will be one of the few randomised controlled trials of psychosocial treatments for this comorbidity to be conducted internationally. The findings are likely to contribute significantly to understanding the types of programs that are effective in treating this comorbidity.

## Abbreviations

ATOS, Australian Treatment Outcome Study; AUC, Area under the receiver operator curve; AU$, Australian dollars; AUDIT, Alcohol Use Disorders Identification Test; BADS-SF, Behavioral Activation for Depression Scale Short Form; BAI, Beck Anxiety Inventory; BATD, Behavioural Activation for Depression; BATD-R, Behavioral Activation for Depression: Revised Treatment Manual; BDI-II, Beck Depression Inventory-II; CIDI 3.0, Composite International Diagnostic Interview version 3.0; CSQ-8, Client Satisfaction Questionnaire; DSM-IV, Diagnostic and Statistical Manual of Mental Disorders-Fourth Edition; DTS, Distress Tolerance Scale; EROS, Environmental Rewards Observation Scale; HREC, Human Research Ethics Committee; IPDE, International Personality Disorders Examination; NHMRC, National Health and Medical Research Council of Australia; OST, Opioid Substitution Therapy; PCL-C, Posttraumatic Stress Disorder Checklist – Civilian Version; PHQ-9, Nine Item Patient Health Questionnaire; PTQ, Perseverative Thinking Questionnaire; PTSD, Post-Traumatic Stress Disorder; RR, Residential Rehabilitation; SCID, Structured Clinical Interview for DSM disorders; SDS, Severity of Dependence Scale; SF-12, Short Form-12 Health Survey; SMAST, Short Michigan Alcohol Screening Test; WHO, World Health Organization.

## Additional files

Additional file 1 “Activate participant information and consent form”—This file provides a model of the information and consent form used in the trial (DOC 293 kb) Additional file 2 “Groups overseeing the trial, insurance coverage provided by UNSW, and authorship guidelines”—This document outlines the role and composition of groups overseeing the trial, the insurance coverage provided by UNSW and the authorship guidelines that will be used for papers based on trial data (DOCX 16 kb)
