# Supplementary material for: The efficacy of behavioural activation treatment for co-occurring depression and substance use disorder (the activate study): a randomized controlled trial
Source: BMC Psychiatry. 2016 Jul 8;16:221. doi: 10.1186/s12888-016-0943-1 (PMC4939012; doi:10.1186/s12888-016-0943-1)
Supplement: Additional file 1 — “Activate participant information and consent form”—This file provides a model of the information and consent form used in the trial (DOC 293 kb) [file 12888_2016_943_MOESM1_ESM.doc]

*
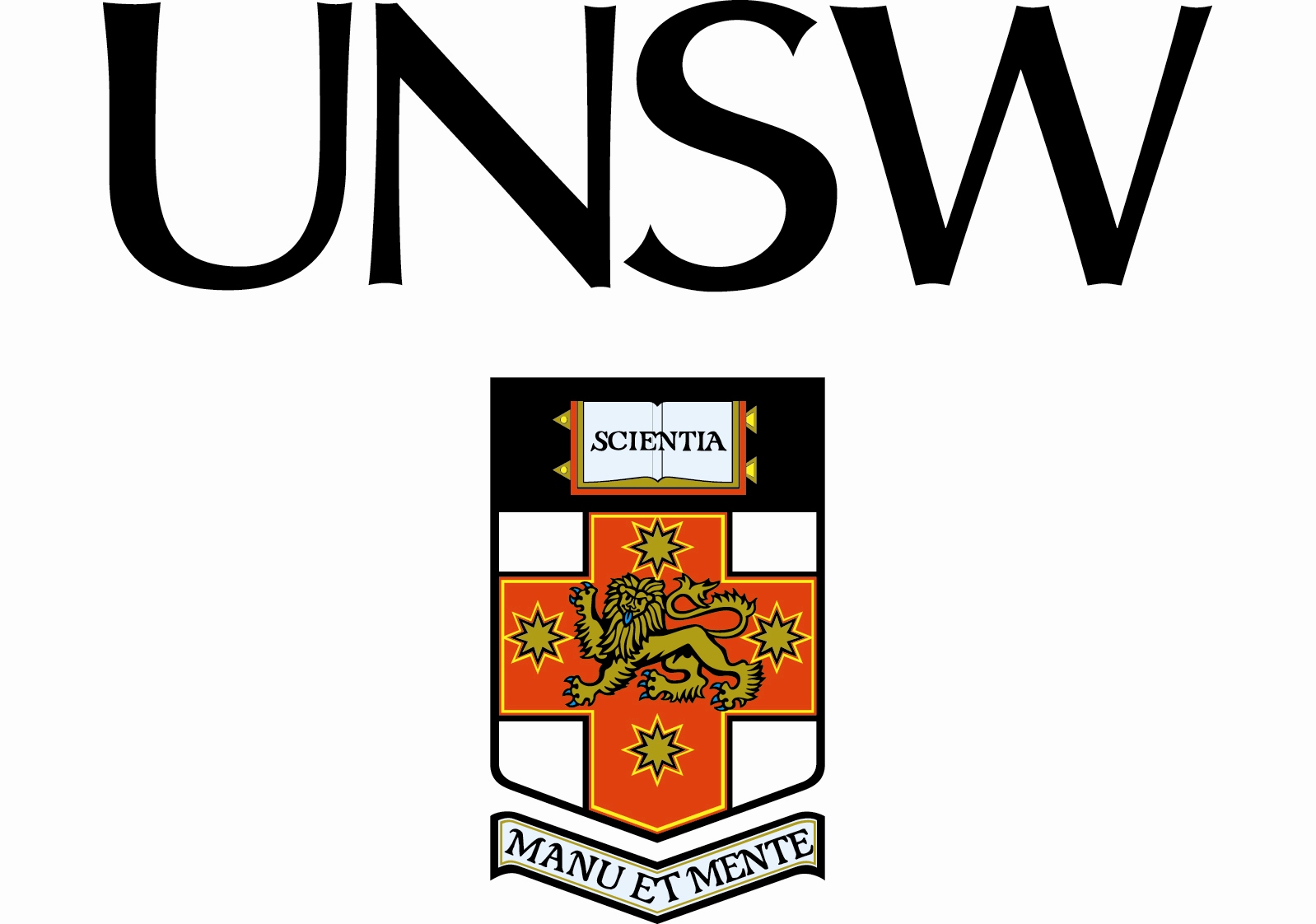
*

# National Drug & Alcohol Research Centre,

# University of New South Wales

Approval No: HC13155

### PARTICIPANT INFORMATION STATEMENT AND CONSENT FORM

**The efficacy of behavioural activation therapy for co-occurring depression and substance use disorder: The Activate Study**

You are invited to participate in a research study examining what works in treating symptoms of depression among people in treatment for drug dependence. We hope to learn whether or not behavioural activation therapy helps to reduce symptoms of depression among individuals in treatment for drug dependence. You were selected as a possible participant in this study because you are currently in treatment for drug dependence and are reporting symptoms of depression.

If you agree to participate in this study, you will be asked to sign the Participant Consent Form.

This study will be conducted over 12 months. On entry to the study, you will be interviewed by the Research Officer on the project about your drug use history, treatment history, symptoms of depression and psychiatric history. This interview will take approximately 60-90 minutes to complete and you will be reimbursed $30 for out of pocket expenses.

The study is a randomised controlled trial comparing standard treatment for drug dependence versus standard treatment combined with individual behavioural activation therapy for the treatment of depression. One hundred participants will be randomly assigned to individual behavioural activation therapy and 100 participants to treatment as usual. Treatment as usual includes opioid replacement therapy or residential rehabilitation treatment. It may also include counselling for depressive symptoms, antidepressant medication where appropriate, and referral to Mental Health when required. To ensure that the two groups (i.e., behavioural activation therapy and treatment as usual) are similar to start with, a computer will allocate each study participant into a group randomly, like the flip of a coin. Neither the therapist nor the study participant can decide which treatment the participant receives.

If you agree to participate in this trial, and you are allocated to individual therapy, you will then be asked to attend ten 1 hour sessions of behavioural activation therapy held weekly. These therapy sessions will be provided by a registered or provisional psychologist. All therapy sessions will be recorded using an MP3 player in order for your psychologist to obtain clinical supervision and gain feedback on how the behavioural activation therapy is being delivered.

Regardless of whether or not you receive individual behavioural activation therapy or treatment as usual, you will also be required to complete interviews at the beginning of your involvement

**PARTICIPANT INFORMATION STATEMENT AND CONSENT FORM (continued)**

**The efficacy of behavioural activation therapy for co-occurring depression and substance use disorder: The Activate Study**

in the study (baseline), and again at 3 and 12 months after baseline. You will be paid $30 per interview for out of pocket expenses. Reimbursement will only be provided for the three interviews and no reimbursement will be provided for attending therapy sessions. The therapy provided as part of this study will not cost you anything.

In order to locate you for follow up interviews, we will need to obtain your contact details and those of 2 or 3 friends or relatives who may be able to assist us in locating you. By signing the consent form you are also granting us permission to locate you through other ways, including social media (e.g., Facebook), Medicare, Centrelink, the Australian electoral rolls, and the pharmaceutical drugs of addictions system (i.e to determine your current dosing point if receiving opioid replacement treatment). Your involvement in the study will remain completely confidential. We would also like your permission to examine your treatment files in order to determine what treatment you received throughout the study period. By signing the consent form you are granting the researchers permission to access your treatment file for this purpose.

Sometimes when people undertake psychological treatment they may initially experience an increase in distress, but this is usually tolerable. The therapists running the programme will be monitoring your mood from week to week, and will offer you further support services if it appears warranted. If you experience significant distress as part of this study you should notify the Project Coordinator (Dr Joanne Ross, Ph. 02 9385 0331).

This study aims to further clinical knowledge and may improve future treatment of co-existing depression and drug dependence. It is hoped that you will experience a reduction in your depressive symptoms. However, we cannot and do not guarantee or promise that you will receive any benefits from this study.

Any information that is obtained in connection with this study and that can be identified with you will remain confidential and will be disclosed only with your permission, except as required by law. If you give us your permission by signing this document, we plan to present the findings of this study at professional seminars, and national and international conferences. We also intend publishing the findings in peer-reviewed journals. Results will also be reported to the Human Research Ethics Committee. Only aggregated group data will be reported and no individuals will be identified. In any publication, information will be provided in such a way that you cannot be identified.

Complaints may be directed to the Ethics Secretariat, The University of New South Wales, SYDNEY 2052 AUSTRALIA (Ph. 9385 4234, Fax 9385 6648, email [ethics.gmo@unsw.edu.au](mailto:ethics.gmo@unsw.edu.au)). Any complaint you make will be investigated promptly and you will be informed of the outcome. On completion of the study, you may obtain a summary of the research findings by contacting one of the research team (Dr Joanne Ross, 9385 0331). Your decision whether or not to participate will not prejudice your future relations with the University of New South Wales or [name of treatment service]*.* If you decide to participate, you are free to withdraw your consent and to discontinue participation at any time without prejudice.

If you have any questions, please feel free to ask us. If you have any additional questions later, Dr Joanne Ross (Ph. 02 9385 0331) will be happy to answer them. You will be given a copy of this form to keep.

THE UNIVERSITY OF NEW SOUTH WALES AND ODYSSEY HOUSE

**PARTICIPANT INFORMATION STATEMENT AND CONSENT FORM (continued)**

**The efficacy of behavioural activation therapy for co-occurring depression and substance use disorder: The Activate Study**

**You are making a decision whether or not to participate. Your signature indicates that, having read the information provided above, you have decided to participate.**

**…………………………………………………… .…………………………………………………….**

Signature of Research Participant Signature of Witness

**…………………………………………………… .…………………………………………………….**

(Please PRINT name) (Please PRINT name)

**…………………………………………………… .…………………………………………………….**

Date Nature of Witness

**REVOCATION OF CONSENT**

**The efficacy of behavioural activation therapy for co-occurring depression and substance use disorder: The Activate Study**

I hereby wish to **WITHDRAW** my consent to participate in the research proposal described above and understand that such withdrawal **WILL NOT** jeopardise any treatment or my relationship with The University of New South Wales, or [name of treatment service].

**…………………………………………………… .…………………………………………………….**

Signature Date

**……………………………………………………**

Please PRINT Name

The section for Revocation of Consent should be forwarded to (Dr Joanne Ross, NDARC, UNSW, Sydney NSW 2052).
