# Supplementary material for: The efficacy of behavioural activation treatment for co-occurring depression and substance use disorder (the activate study): a randomized controlled trial
Source: BMC Psychiatry. 2016 Jul 8;16:221. doi: 10.1186/s12888-016-0943-1 (PMC4939012; doi:10.1186/s12888-016-0943-1)
Supplement: Additional file 2 — “Groups overseeing the trial, insurance coverage provided by UNSW, and authorship guidelines”—This document outlines the role and composition of groups overseeing the trial, the insurance coverage provided by UNSW and the authorship guidelines that will be used for papers based on trial data (DOCX 16 kb) [file 12888_2016_943_MOESM2_ESM.docx]

**Composition, roles, and responsibilities of the coordinating centre, steering committee, endpoint adjudication committee, data management team, and other individuals or groups overseeing the trial, if applicable**

Coordinating Centre – NDARC, MT, JR, KM & SK

- Design and conduct of the Activate Study
- Preparation of protocol and revisions
- Preparation of participant questionnaires
- Organising steering committee meetings
- Publication of study reports

Steering Committee (SC) - MT, JR, CL, KM, SK, KB & GD

- Agreement of final protocol
- Reviewing progress of study

Trial management committee (TMC) - MT, JR, & KM

- Study planning
- Provision of annual ethics committee reports
- Budget administration
- Data verification
- Randomisation

Data management Team – JR, KP, XL, JC, PE, SM & SLM

- Maintenance of database and data entry
- Data verification

**Composition of data monitoring committee (DMC); summary of its role and reporting structure; statement of whether it is independent from the sponsor and competing interests; and reference to where further details about its charter can be found, if not in the protocol. Alternatively, an explanation of why a DMC is not needed**

As the risks associated with behavioural activation therapy are deemed to be minimal, and the therapy is of short duration (10 one hour sessions), no data monitoring committee was deemed necessary.

**Provisions, if any, for ancillary and post-trial care, and for compensation to those who suffer harm from trial participation**

In the unlikely event that a participant suffers harm from trial participation, the Activate Study is covered by UNSW’s Clinical Trials Insurance Policy, which provides cover for human clinical trials, medical malpractice, and no fault compensation for UNSW clinical trials. This includes cover for additional health care, compensation or damages.

**Authorship eligibility guidelines and any intended use of professional writers**

The research team will adhere to the BMJ authorship guidelines (<http://www.bmj.com/about-bmj/resources-authors/article-submission/authorship-contributorship>) in determining authorship on manuscripts. The team do not intend using professional writers.
